# Gender minority stress, resilience, and mental health in clinic-referred transgender and gender-diverse adolescents: a network analysis

**DOI:** 10.1186/s13034-026-01031-6

**Published:** 2026-01-29

**Authors:** Aisa Burgwal, Anna I. R. van der Miesen, David Matthew Doyle, Annelou L. C. de Vries

**Affiliations:** 1 Department of Child and Adolescent Psychiatry, Centre of Expertise on Gender Dysphoria, Amsterdam UMC, Amsterdam, The Netherlands; 2 Department of Medical Psychology, Amsterdam UMC, Amsterdam, The Netherlands

**Keywords:** Transgender and gender-diverse youth, Gender minority stress, Mental health, Resilience, Network analysis

## Abstract

**Background:**

Transgender and gender-diverse (TGD) adolescents experience elevated rates of depression, anxiety, and suicidality compared to cisgender peers. These outcomes are largely hypothesized to be linked to social stigma and gender minority stress. Resilience constructs like identity pride and community connectedness may buffer these stressors, but their role, especially in clinical populations, remains underexplored. This study investigates the interplay between gender minority stress, resilience, and mental health in clinic-referred Dutch TGD youth through network analysis.

**Methods:**

A sample of 172 Dutch TGD adolescents was assessed using commonly employed measures of gender minority stress, resilience, and mental health. Network analysis was conducted to examine network structure and centrality of these constructs. Among the 172 clinic-referred Dutch participants (mean age = 15.70, *SD* = 0.79), 86.0% (*n* = 148) were assigned female at birth, 14.0% (*n* = 24) were assigned male at birth, and 9.9% (*n* = 17) of the total sample identified as non-binary and genderqueer (NBGQ).

**Results:**

Among the participants, 41.7% scored within the clinical range for internalizing problems, and 32.7% exhibited scores indicative of mild to severe depression. Non-affirmation, an established construct in gender minority stress literature, emerged as the most central construct in the network, with strong associations to internalized transphobia and negative expectations for future events. Distal gender minority stressors, such as gender-related rejection and victimization, were less central within the network, but indirectly influenced mental health via pathways involving the more central stressors. Resilience constructs, specifically pride and community connectedness, showed limited centrality.

**Conclusions:**

The findings highlight the substantial psychological vulnerabilities of Dutch TGD adolescents and underscore the importance of addressing non-affirmation, internalized transphobia, and negative expectations for future events in clinical settings to mitigate mental health challenges among TGD youth. The study also emphasizes the necessity of societal-level interventions, such as raising awareness about the value of affirming gender identity. Future research should explore the nuanced distinctions within and between different gender minority stressors and their specific impacts on TGD youth.

**Supplementary Information:**

The online version contains supplementary material available at 10.1186/s13034-026-01031-6.

## Background

Transgender and gender-diverse (TGD) adolescents represent a population whose gender identities and/or expressions do not conform to the culturally defined norms associated with their sex assigned at birth [1]. During adolescence and adulthood, TGD individuals encounter a disproportionate degree of mental health challenges, such as depression, anxiety, and suicidality, compared to cisgender populations [2–8]. These poor mental health outcomes are often attributed to stigma-related stress in various forms, such as experiences of bullying, discrimination, and victimization, internalized transphobia, and non-affirmation of gender identity [9–12].

Social stigma (i.e., societal disapproval or discrimination towards a group based on their perceived differences that set them apart from other members of society [13]) is now increasingly recognized as a driver of mental health disparities among TGD people. Minority Stress Model research shows that sexual and gender minority individuals face unique, chronic stressors that harm their well-being [9, 14, 15]. New evidence finds that TGD individuals experience even higher levels of minority stress than lesbian, gay, and bisexual populations [10, 16–20]. The Minority Stress Model delineates two distinct categories of social stressors [21]. Distal stressors, such as discrimination and rejection, constitute external stressors. Proximal stressors, related to internalized stress, encompass the fear of future victimization and internalized transphobia [9, 22]. Compared to other groups within the Lesbian, Gay, Bisexual, Transgender, Intersex, and more (LGBTI+) community, research consistently shows that TGD individuals experience distal stressors at disproportionally high rates [23]. A comprehensive study across all European Union Member States recorded that 48% of TGD individuals, aged 15 and older, reported experiencing identity-related harassment within the past year, and 17% reported having been subjected to physical and/or sexual assaults in the previous five years [23]. The study highlighted that younger age groups (15–17 years) were particularly vulnerable. TGD individuals can internalize (i.e., deeply absorb) these external stressors, which can lead to the emergence and/or increase of internalized/proximal stressors [9, 22]. Internalization refers to the process of absorbing messages, which can lead to self-deprecation, self-hatred, and a belief that the messages are true [24]. For example, Perez-Brumer et al. [25] demonstrated that internalized transphobia, which refers to the internalization of negative societal attitudes regarding one’s gender identity, significantly heightens the risk of lifetime suicide attempts among transgender adults. There is a robust association between distal and proximal stressors within the TGD community (e.g., 26, 27, 28). For example, TGD individuals may grapple with negative self-perceptions due to past rejection and discrimination [29]. To navigate societal expectations, they sometimes conceal their gender identity when in public [19, 24, 29]. Another unique stressor for TGD individuals is non-affirmation of gender identity, which refers to interactions with environments that do not recognize or value their gender expression and internal sense of gender identity [10, 30].

In addition to gender minority stressors, various studies highlight the potential protective impact of resilience factors [31, 32]. These factors include identity pride and community connectedness, which may mitigate the adverse effects of minority stress on mental health and well-being of TGD individuals [9, 22]. Research indicates that TGD youth can cope with minority stressors when resilience factors, such as access to social support, are present to facilitate adaptive coping mechanisms [32, 33]. However, findings on resilience factors beyond social support among TGD youth remain underexplored.

In recent years, the Gender Minority Stress and Resilience (GMSR) measure has been developed and validated for TGD adults [10]. Subsequent studies using the GMSR measure have found positive associations between gender minority stress and symptoms of anxiety and depression [34–37]. These studies additionally found that proximal stressors are more strongly associated with mental health outcomes than distal stressors [38], and that resilience factors are associated with lower levels of anxiety and depression in TGD individuals [38, 39].

Compared to TGD adults, there is a notable lack of studies on the impact of gender minority stressors and resilience on TGD youth. To date, the GMSR measure remains the only validated instrument specifically designed to measure these constructs with gender minority populations. Although originally developed and validated in English for adults, it has been increasingly applied to youth populations in recent studies. In some initial work, Chavanduka et al. [33] identified differences among various TGD youth groups using the GMSR measure, demonstrating its utility despite focusing primarily on cohort variations rather than individual stressors’ associations with mental health. Other recent studies have explored these associations. For example, Miller-Perusse et al. [28] found that greater distal stress is associated with less resilience, which in turn is associated with increased psychological distress in TGD youth aged 15 to 24. Similarly, Dolezal et al. [27] reported that higher distal stress correlates with more post-traumatic stress disorder symptoms and anxiety symptoms, and that distal stress indirectly predicts depression via proximal stress. However, the precise roles of gender minority stressors and resilience, and their interrelations with mental health in TGD youth, remain unclear. In the current study, the Dutch translation of the GMSR measure was used. Although this version has not yet been formally validated, it was carefully translated and is used here due to the absence of alternative validated tools in Dutch.

Therefore, the primary objective of this study was to investigate the associations between gender minority stress, resilience and mental health among TGD adolescents. Mental health was operationalized through internalizing symptoms (e.g., depression), in line with prior research identifying these as key indicators of psychological distress in TGD youth [27, 28, 40, 41]. We applied network analysis to examine the interrelations between GMSR-measured constructs and symptom scales for depression, internalizing problems, and suicidality. By doing so, we can recognize clusters of stressors that tend to co-occur, providing a detailed picture of the symptomatology and stress experiences of TGD youth. No prior network studies have investigated these constructs within the Netherlands, despite Dutch TGD youth functioning relatively well compared to peers in other Western countries (i.e., Belgium, Germany, the UK, Canada) [42, 43]. This is likely due to higher levels of social acceptance [42], although structural barriers and stigma persist to exist [44]. Investigating these dynamics within a Dutch sample therefore offers a unique opportunity to explore how minority stress and resilience manifest within a comparatively affirming context, informing sensitive clinical practices and interventions.

## Methods

### Participants and study design

The sample for this study consisted of consecutive referrals to the Center of Expertise on Gender Dysphoria (Amsterdam UMC) between July 5, 2022, and March 18, 2025. Adolescents aged 13 to 17, who identified as TGD, underwent a comprehensive psychodiagnostic assessment, including the questionnaires used for this study (see below). Out of 194 adolescents, 172, along with their parents/legal guardians, provided informed consent for the use of their data for scientific purposes. The study received ethical approval by the Medical Ethics Review Committee of Amsterdam UMC.

The series of questionnaires included items related to sociodemographic information (i.e., sex assigned at birth, gender identity, age, educational level), gender minority stress and resilience, and mental health. Gender identity was assessed using a multiple-response question which asked participants to select the gender(s) that best described them (options included *transgender*,* girl*,* boy*,* non-binary*,* agender*,* investigating*, and *other*). TGD youth were categorized based on a combination of their self-identified gender and their sex assigned at birth. Adolescents who were assigned female at birth and identified as *transgender* and/or *boy* were categorized as trans masculine, while assigned male at birth who identified as *transgender* and/or *girl* were categorized as trans feminine. *Non-binary* and *agender participants* were recoded as non-binary and genderqueer (NBGQ). Participants who selected *investigating* and/or *other* were each reviewed to determine if they identified with a NBGQ gender identity (i.e., *non-binary*, *agender*) or only with a binary gender identity (i.e., *boy*, *girl*) and were all recoded accordingly. Educational level was coded as *vocational education* and *higher vocational education* in line with previous research [45].

### Main outcome measures

#### Gender minority stress and resilience

The experience of gender minority stress and resilience was assessed using the Dutch translation of the Gender Minority Stress and Resilience (GMSR) measure [10]. The scale includes four distal minority stress constructs (i.e., gender-related discrimination, gender-related rejection, gender-related victimization, non-affirmation of gender identity), three proximal minority stress constructs (i.e., internalized transphobia, negative expectations for future events, nondisclosure), and two resilience constructs (i.e., community connectedness, identity pride; 10). For an overview of the GMSR subscales, see Table 1. To improve the reliability of the measurement for the TGD youth sample, we evaluated the Cronbach’s alpha of each subscale. Items were removed when their deletion resulted in an increase in the Cronbach’s alpha of the total subscale. Specifically, within the gender-related discrimination subscale, item 2 (*“Because of my gender identity or expression*,* I have had difficulty finding a bathroom to use when I am out in public”*) and item 3 (*“I have experienced difficulty getting identity documents that match my gender identity”*) were excluded. Within the gender-related rejection subscale, item 2 (*“I have been rejected or made to feel unwelcome by a religious community because of my gender identity or expression”*) and item 6 (*“I have been rejected or distanced from family because of my gender identity or expression”*) were removed. For the gender-related victimization subscale, item 2 (*“I have been threatened with being outed or blackmailed because of my gender identity or expression”*) was deleted. Finally, within the community connectedness subscale, item 4 (*“I’m not like other people who share my gender identity”*) and item 5 (*“I feel isolated and separate from other people who share my gender identity”*) were excluded.

For the distal stressors, response options for gender-related discrimination, rejection, and victimization are “never”, “yes, before age 18”, “yes, after age 18”, “yes, in the past year”, and “not applicable”. For our sample (13–17 years of age), the response option “yes, after age 18” was not used. Responses were dichotomized (0 = no event, 1 = any event) [10], and scale scores reflect the sum of endorsed events of gender-related discrimination, rejection, and victimization. Response options for non-affirmation, the proximal constructs (i.e., internalized transphobia, negative expectations for future events, and non-disclosure), and the resilience constructs (i.e., pride and community connectedness) ranged from “0 = strongly disagree” to “4 = strongly agree”. Item scores were summed to give the score of each construct. For the distal and proximal constructs, higher values indicate greater levels of stress, whereas for the resilience constructs, higher values indicate greater levels of resilience [10]. All nine constructs from the GMSR measure were used. In summary, gender-related discrimination, rejection, and victimization are count variables, whereas non-affirmation, the proximal constructs, and resilience constructs are scale variables.

**Table 1:** Gender minority stress and resilience measure

Scale	# of used items	Reported scale range	Cronbach’s α	# of original items	Original scale range (11)	Response options	Example item
Distal stress	17	0–46		24	0–60		
Gender-related discrimination	3	0–6	.56	5	0–10	*“never”, “yes, before age 18”, “yes, in the past year”, “not applicable”*	*“I have had difficulty finding employment or keeping employment, or have been denied promotion because of my gender identity or expression”*
Gender-related rejection	4	0–8	.52	6	0–12	[see discrimination]	*“I have been rejected at school or work because of my gender identity or expression”*
Gender-related victimization	4	0–8	.73	7	0–14	[see discrimination]	*“I have been threatened with physical harm because of my gender identity or expression”*
Non-affirmation of gender identity	6	0–24	.91	6	0–24	*“0 = strongly disagree” – “4 = strongly agree”*	*“I have difficulty being perceived as my gender”*
Proximal stress	22	0–88		22	0–88		
Internalized transphobia	8	0–32	.89	8	0–32	[see non-affirmation]	*“I resent my gender identity or expression”*
Negative expectations for future events	9	0–36	.93	9	0–36	[see non-affirmation]	*“If I express my gender identity/history, I could be denied good medical care”*
Gender identity/history non-disclosure	5	0–20	.76	5	0–20	[see non-affirmation]	*“Because I don’t want others to know my gender identity/history, I modify my way of speaking”*
Resilience factors	11	0–44		13	0–52		
Pride	8	0–32	.86	8	0–32	[see non-affirmation]	*“It is a gift that my gender identity is different from my sex assigned at birth” *
Community connectedness	3	0–12	.86	5	0–20	[see non-affirmation]	*“When interacting with members of the community that share my gender identity, I feel like I belong”*

*Cronbach’s α of the final subscale used for this study is reported in the Table. *

#### Mental health: internalizing problems, suicidality, and depression

##### Youth Self-Report (YSR)

The Dutch version of the YSR was used to assess internaling problems and thoughts of self-harm and suicidality [46]. The YSR, consisting of 118 items on a 3-point scale, evaluates internalizing and externalizing problem behaviors and has strong reliability and validity [46]. This study focused on internalizing problems (α = 0.91 in this study sample) and self-harm/suicidality (α = 0.81). The internalizing problems scale, comprised of 31 items, yields a total sum score ranging from 0 to 62, which is then transformed into *T-*scores. *T*-scores above 63 are considered to be in the clinical range, while 60–63 are borderline clinical range scores. About 10% of Dutch adolescents from the general population sample score in the clinical range [47]. Self-harm/suicidality was examined using two items: *“I deliberately try to hurt or kill myself”* and *“I think about killing myself”*. As done in previous studies, the composite score for these two items was calculated [48]. Notably, the first item of the self-harm/suicidality scale is also part of the internalizing problems scale.

##### Beck Depression Inventory II (BDI-II)

In addition, the participants completed the Dutch translation of the BDI-II. This is a 21-item inventory on a 3-point scale, measuring presence and degree of depression symptoms in adolescents and adults, with good psychometric properties (α = 0.92 in this study sample) [49, 50]. It consists of items such as *“I don’t feel particularly guilty”*,* “I feel I may be punished”*, and* “I find I can’t concentrate on anything*”. A total score between 14 and 19 is suggestive of a mild depression, a score between 20 and 28 of a moderate depression, and a score of more than 29 of a severe depression.

### Statistical analysis

All analyses were conducted using R (Version 4.4.0) [51]. Descriptive statistics, including frequencies, proportions, means (*Ms*), and standard deviations (*SD*s), were calculated to summarize the demographic characteristics of the sample. Subsequently, the *M*s and *SD*s of all GMSR, YSR, and BDI-II item scores were examined to compare the GMSR constructs to each other and to analyze the proportions of clinical range scores of internalizing problems and depression. As participants were required to complete all items on the GMSR, YSR, and BDI-II, no missing data were present, and analyses were therefore conducted on complete cases only.

To address the research questions, network analysis was employed to examine associations between the GMSR constructs and the BDI-II/YSR scales. Pearson correlation coefficients were used to assess relationships between measures. The ratio between sample size (*N* = 172) and number of nodes (*n* = 12) in the current study is favorable, providing sufficient observations per node to support stable estimation of edge weights and centrality indices [52]. Nevertheless, given the relatively modest sample size, a graphical least absolute shrinkage and selection operator (LASSO) method was used for regularization of the network structure [52, 53]. The regularization parameter (gamma) was set at default, which proved to be a good trade-off between model complexity and goodness-of-fit. Extended Bayesian Information Criterion (EBIC) was applied to the graphical LASSO method for model selection. EBIC regularization helps balance model complexity and goodness of fit, resulting in a sparse network model that is more interpretable than the original model [54, 55].

To estimate and visualize the network, R packages “qgraph” (Version 1.9.8) [56] and “bootnet” (Version 1.6) [52] were used. Each node (representing a GMSR construct or a mental health outcome scale) is connected to a range of other nodes through edges with different weights [57]. The network is visualized using a force-directed graph layout algorithm [56]. The predictability of each node was estimated using the function “estimateNetwork” from the R-package “bootnet” (Version 1.6) [52]. Predictability refers to how well each node can be predicted by neighboring nodes in the network.

Network analysis can also provide quantitative centrality indicators for each node based on the unique configuration of the network. As indicated by prior research [52, 58], the centrality index of strength was used to denote the significance of individual nodes in the model. Certain other indices of centrality, such as betweenness and closeness, have been shown to be unsuitable in psychological symptom networks and were therefore not calculated [59]. Strength centrality refers to the absolute sum of edge weights connecting one node to all other nodes, with higher values reflecting greater centrality in the network. Following previous studies [60, 61], bridge strength, the best index for identifying nodes in which deactivation of a groups of stressors (e.g., distal stressors) could prevent activation of another stressor (e.g., proximal stressors, internalizing problems) was used to identify bridge nodes. To test bridge strength, we first classified our clusters based on the Minority Stress Model. We clustered all distal stressors (i.e., gender-related discrimination, gender-related rejection, gender-related victimization, and non-affirmation), proximal stressors (i.e., internalized transphobia, negative expectations for future events, and gender identity/history non-disclosure), resilience constructs (i.e., pride and community connectedness), and mental health outcomes (i.e., BDI-II scores, YSR Internalizing and YSR Suicidality scores). Building on this foundation, bridge strength was employed as a metric to discern which nodes serve as pivotal connectors between distal stressors, proximal stressors, resilience, and mental health outcomes. The reported bridge strength values were standardized to range from zero to one, representing the relative strength of node connections across clusters.

*Assessment of network accuracy and stability.* Following Epskamp et al. [52], the robustness of the network solution was evaluated by estimating edge weight accuracy and centrality index stability. Edge weight accuracy was determined through non-parametric bootstrapping with 1000 samples to compute 95% confidence intervals (CIs) [62]. Wider CIs indicated lower precision while narrower CIs indicated higher accuracy of the network [52]. Correlation stability coefficients (CS-C) evaluated the stability of centrality indices through subset bootstraps [63]. CS-C values represent the maximum proportion of samples that could be removed while maintaining a 95% probability that the correlation between original and bootstrapped centrality indices would be at least 0.70. Ideally, the CS-C should be above 0.50, and not below 0.25 [52].

## Results

### Participants

Of the 172 participants included in the final sample, the majority of participants were female assigned at birth (86.0%, *n* = 148), 14.0% (*n* = 24) assigned male at birth, and 9.9% identified as non-binary and genderqueer (NBGQ, *n* = 17). Mean age of the sample was 15.7 (*SD* = 0.79, range = 13–17). Demographic characteristics of the study sample are presented in Table 2.

**Table 2:** Demographic characteristics of the study sample

Variables	
Age, *M* (*SD*)	15.7 (0.79)
Sex assigned at birth, *n* (%)	
Female	148 (86.0)
Male	24 (14.0)
Gender identity, *n* (%)	
Feminine	23 (13.4)
Masculine	132 (76.7)
NBGQ	17 (9.9)
Education, *n* (%)	
Vocational education	47 (35.9)
Higher vocational education	76 (58.0)
Don’t go to school	8 (6.1)

*The total n for each of the demographic variables depends on the availability of the data in the sample. NBGQ = non-binary and genderqueer*,* M = mean*,* SD = standard deviation*,* n = sample size*

### Distributions and severity of mental health symptoms and minority stressors among TGD youth

Mean raw/*T*-scores, *SDs*, and ranges of all the GMSR constructs, the BDI-II, and YSR scales are presented in Table 3. The analysis revealed that 41.7% (*n* = 55) of the sample scored within the clinical range for YSR internalizing problems (*T*-score above 63), and 12.9% (*n* = 17) scored within the borderline range (*T*-score between 60 and 63). The proportion of youth scoring in the clinical range (41.7%) substantially exceeds the 10% observed in the Dutch general population standardization sample [47]. Out of the total sample, 32.7% (*n* = 55) had BDI-II scores within the range indicative of mild to severe depression. Specifically, 11.9% (*n* = 20) of these participants exhibited mild depressive symptoms, 14.3% (*n* = 24) demonstrated moderate depressive symptoms, and 6.5% (*n* = 11) were classified as having severe depressive symptoms. The analysis of the GSMR measure revealed that mean counts on the three distal stressors (i.e., gender-related discrimination, rejection, and victimization) were relatively low (see Table 3 for the mean scores). For example, the mean count for gender-related discrimination is 0.73 with a maximum count of 6, indicating that, on average, participants reported experiencing fewer than one event of gender-related discrimination. Non-disclosure scores, which reflect poorer outcomes when higher, emerged as the most elevated among the GMSR constructs, with community connectedness ranking second highest.

**Table 3:** Descriptive statistics of the GMSR constructs, BDI-II, YSR Internalizing, and YSR suicidality

Scale	M	SD	Scale range
Distal stress			
Gender-related discrimination	0.73	1.11	0–6
Gender-related rejection	1.05	1.34	0–8
Gender-related victimization	1.60	1.71	0–8
Non-affirmation	1.70	1.08	0–4
Proximal stress			
Internalized transphobia	1.44	0.92	0–4
Negative expectations for future events	1.27	0.83	0–4
Gender identity/history non-disclosure	2.29	0.85	0–4
Resilience			
Pride	1.69	0.80	0–4
Community connectedness	2.25	0.98	0–4
BDI-II (total raw score)	11.30	10.43	0–63
YSR-Internalizing (*T*-score)	59.97	11.19	
YSR-Suicidality (total raw score)	0.23	0.46	0–2

*M = Mean, SD = Standard deviation*

### Network structure and centrality measures analysis

**Fig. 1:**
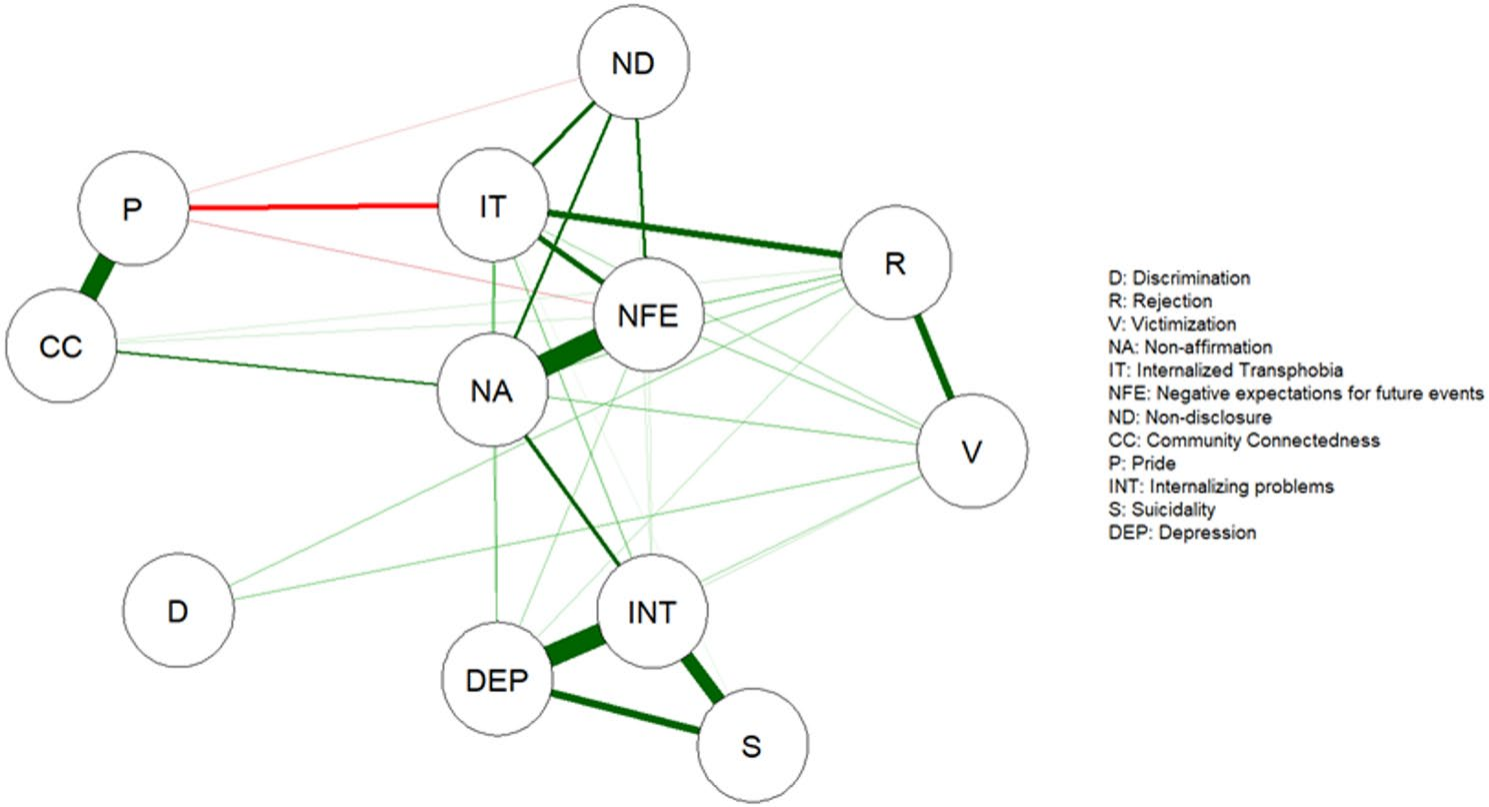
Network structure of mental health outcomes, gender minority stress and resilience. Note: In the network, nodes with stronger and more frequent connections are closer to each other. The dark green lines represent positive associations, the red lines negative associations. The edge thickness represents the strength of the associations between symptom nodes

Figure 1 illustrates the network analysis of the GMSR measure, the BDI-II, the YSR Internalizing problems, and the YSR Suicidality scale using the EBICglasso model [54, 55]. Various nodes displayed differing edge weights (i.e., associations with other nodes), thereby contributing to the overall understanding of the network dynamics. The analysis revealed that the GMSR construct *negative expectations for future events* (NFE) had the strongest connection with GMSR construct *non-affirmation* (NA) followed by the connection between *depression* (DEP) and *internalizing problems* (INT), and the connection between *internalizing problems* (INT) and *suicidality* (S). All mental health scales demonstrated strong interconnections (DEP, INT, and S). Conversely, *gender-related discrimination* (D) showed minimal associations with other nodes in the model, with only limited connections to *gender-related victimization* (V) and *gender-related rejection* (R) nodes, indicating a marginal influence within the network. Similarly, resilience nodes *pride* (P) and *community connectedness* (CC) demonstrated a strong positive association with each other, but showed relatively limited connections with other nodes in the model. However, *pride* (P) showed a strong negative association with node *internalized transphobia* (IT), while node *community connectedness* (CC) demonstrated a positive link with node *non-affirmation* (NA), indicating that although they are somewhat integrated in the model, their overall connectivity remains modest. Regarding the directions of associations, most edges between nodes were positive, indicating that more distal stress is associated with more proximal stress (or vice versa), and both are linked to increased mental health symptoms.

**Fig. 2:**
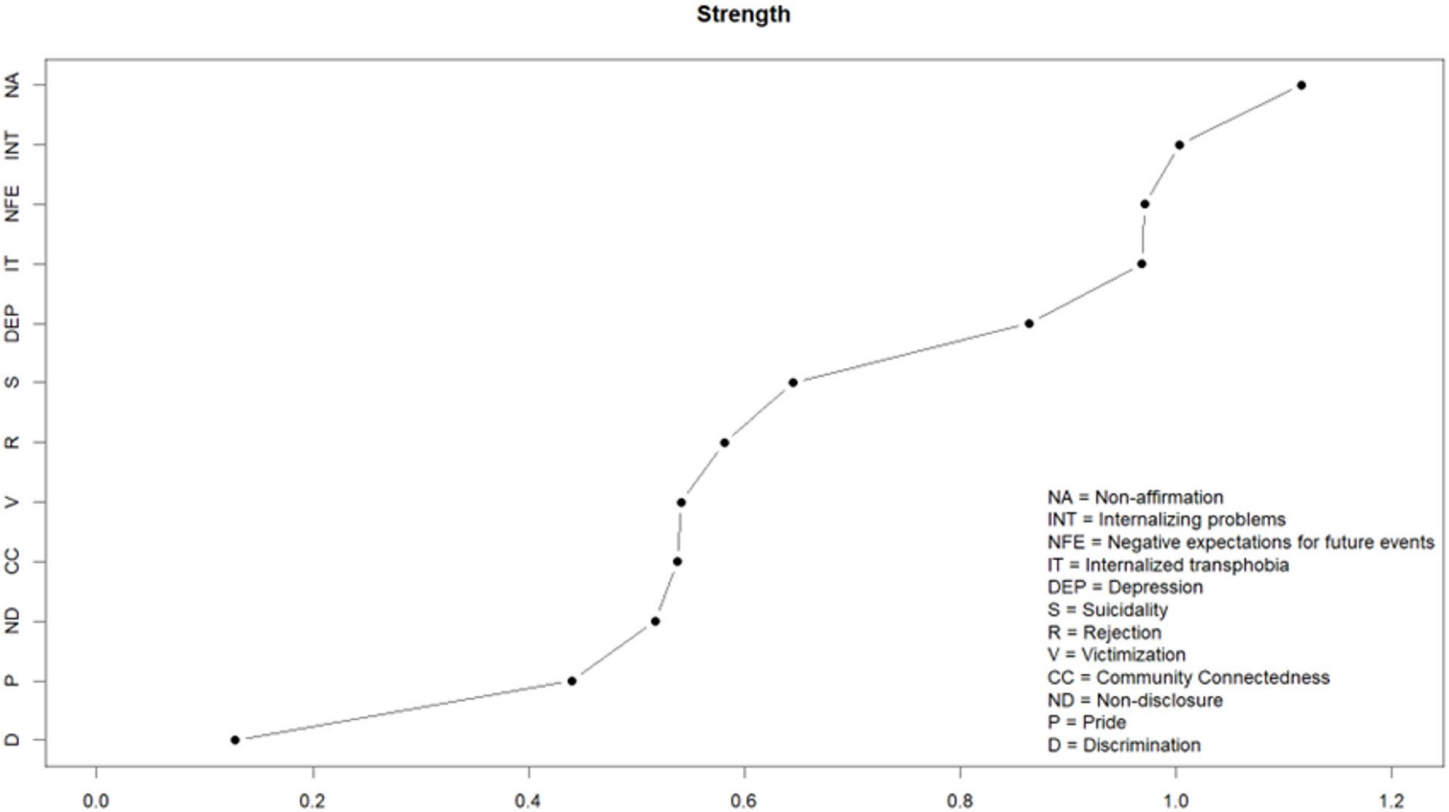
Node strength centrality of the mental health outcomes, gender minority stress and resilience

In terms of node strength centrality, which indicates the influence and connectivity of a node with other nodes within a network, the node representing *non-affirmation* (NA) had the highest strength centrality. Similarly, GMSR constructs *negative expectations for future events* (NFE) and *internalized transphobia* (IT) also had greater strength centrality than most other nodes in the network (see Fig. 2). Consequently, these three GMSR constructs can be seen as central to understanding the network of gender minority stressors, resilience, and mental health in this sample. In contrast, several other scales, including nodes *community connectedness* (CC), *non-disclosure* (ND), *pride* (P), and *gender-related discrimination* (D), were less central and thus positioned on the exterior of the network.

**Fig. 3:**
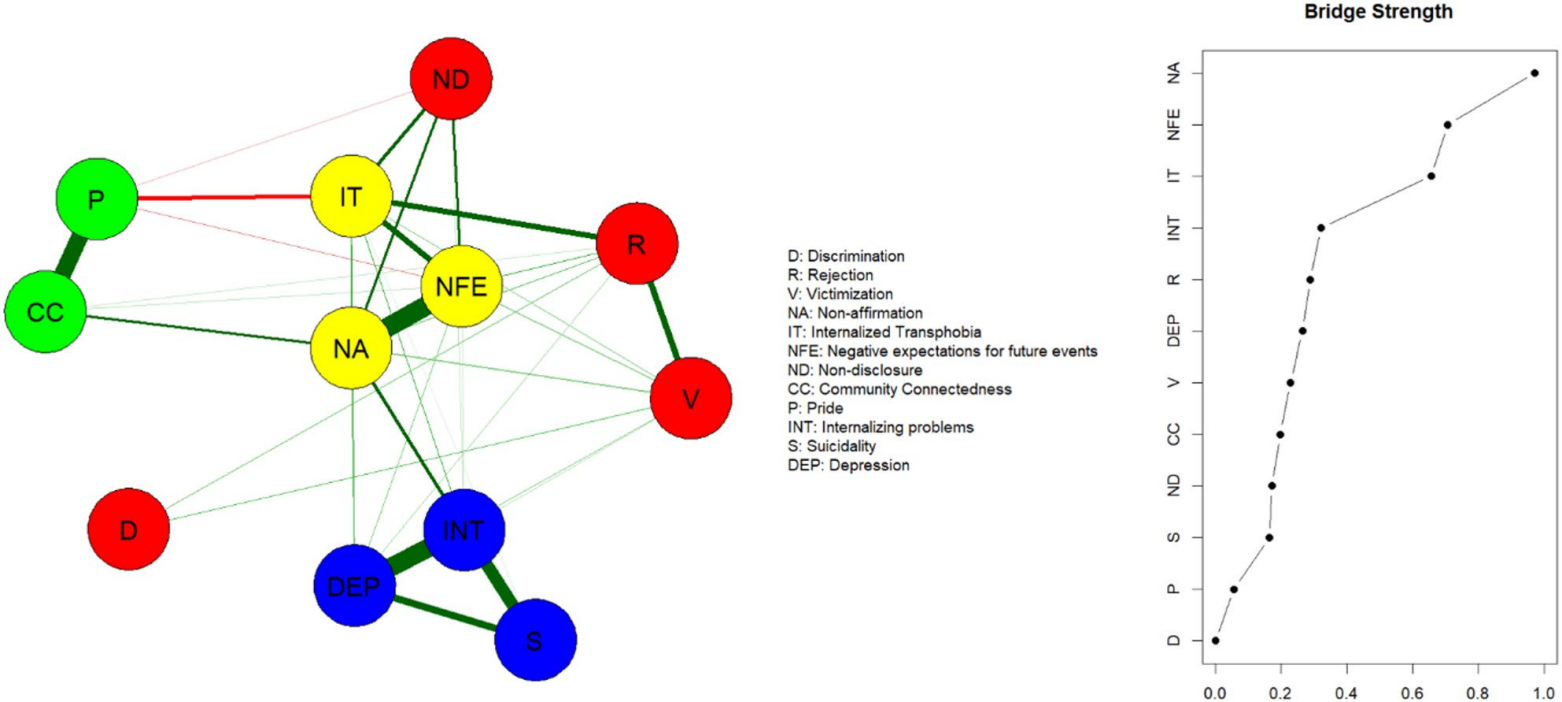
Network structure of mental health outcomes, gender minority stress and resilience showing bridge nodes. Note: In the network, nodes with stronger and more frequent connections are closer to each other. The yellow nodes denote the bridge nodes (non-affirmation, negative expectations for future events, internalized transphobia); the red nodes denote the other gender minority stress constructs, the green nodes denote the resilience constructs; the blue nodes denote the mental health outcomes (depression, internalizing problems and suicidality). The dark green lines represent positive associations, the red lines negative associations. The edge thickness represents the strength of the association between nodes

As illustrated in Fig. 3, Nodes *non-affirmation* (NA), *negative expectations for future events* (NFE), and *internalized transphobia* (IT) emerged as the three most prominent bridge nodes. These nodes potentially play critical bridging roles in linking other GMSR constructs and mental health outcomes. Acting as connectors within the network, these three nodes facilitate connections between different constructs. The deactivation of each of these stressors could potentially prevent the activation of other stressors and their possible impact on mental health outcomes. For instance, the node *non-disclosure* (ND) demonstrates positive associations with the nodes *internalized transphobia* (IT), *non-affirmation* (NA), and *negative expectations for future events* (NFE). By specifically addressing or mitigating the bridge nodes IT, NA, and NFE, it may be possible to disrupt the pathways through which the proximal stressor non-disclosure, among others, influences mental health, thereby reducing the overall burden on mental health outcomes.

A correlation stability analysis was performed to calculate the correlation stability coefficient (CS-C), which denotes the estimated maximum number of cases that can be dropped from the data to retain, with 95% probability, a correlation of at least 0.70 between the original network statistics bootstrapped resamples. For stability of the network analysis, strength centrality had a moderate level of stability (i.e., CS-C = 0.285), indicating that 28.5% of the sample could be dropped without significant changes. Bootstrapped 95% CIs for estimated edge weights suggested that the estimates were reliable and stable (see Additional file 1).

## Discussion

This study represents the first evaluation of the association between gender minority stress and resilience, as measured by the GMSR measure, and mental health, as measured by the BDI-II and the YSR Internalizing and Suicidality scales, among clinic referred Dutch transgender and gender-diverse (TGD) adolescents. Using a network approach, we examined how these gender minority stressors constitute a psychological network and vary in their centrality and strength within that network. Our findings demonstrated that both distal and proximal stressors are associated with depression, internalizing problems, and suicidality. This aligns with previous research indicating that increased gender minority stress is associated with heightened mental health challenges [27, 28, 38]. Notably, the construct of non-affirmation exhibited both the highest strength centrality within the network, and numerous strong connections with other constructs, such as negative expectations for future events, non-disclosure, and internalizing problems. Similarly, negative expectations for future events and internalized transphobia also showed high node strength centrality, suggesting all three constructs are central and well-connected in the network.

Distal stressors, such as gender-related rejection, gender-related victimization, and non-disclosure, showed lower strength centrality and were positioned on the periphery of the network. Consistent with previous research [26–28], these distal stressors may still play a role indirectly through proximal stressors, such as internalized transphobia and negative expectations for future events. Gender-related discrimination exhibited the lowest strength centrality in this sample. On the one hand, this may suggest a weaker association between discrimination and other minority stressors or mental health outcomes. On the other hand, this discrepancy may also reflect limitations in the measurement of the discrimination construct, which includes items less applicable to 13–17-year-olds in the Netherlands (e.g., employment issues, issues finding housing). As the GMSR measure was originally developed for adults, some items may not adequately capture adolescent experiences, and it remains unclear whether adapting these items would significantly alter associations in the network. Additionally, discrimination rates for TGD youth may simply be lower in the Netherlands compared to other studies conducted at the European level [23].

Non-affirmation of gender identity has predominantly been considered a distal stressor in earlier research [10, 64, 65]. However, our findings indicated that non-affirmation had the highest node strength and the highest bridge strength centrality, suggesting it is both highly influential within the network and serves as a key construct linking both distal and proximal stressors. This pattern underscores that non-affirmation may be a unique gender minority stressor that occupies an intermediate position between distal and proximal stressors, being more externally influenced than internalized transphobia but not as directly related to external actions as gender-related discrimination. However, conceptual and measurement considerations must be acknowledged. Within the GMSR measure, non-affirmation items seem to capture actions taken by TGD individuals to minimize or prevent experiences of non-affirmation (e.g., managing gender expression), rather than the direct external stressor of being non-affirmed [66]. As a result, these items may reflect coping or avoidance behaviors rather than external stress exposure, which may explain why non-affirmation occupies an intermediate position in the network. Furthermore, prior research has demonstrated that peer and school contexts play an important role in identity development [67, 68], contexts where affirmation of gender identity is challenged on a daily basis [69, 70]. Within these settings, experiences of non-affirmation can impact mental health outcomes [69], a finding that aligns with the centrality of non-affirmation in our network. We recommend future studies differentiate the gender minority stressor non-affirmation since it seems to play such a central role in relation to other gender minority stressors and in relation to the mental health of Dutch TGD youth. However, it is important to consider that gender minority stress might work differently in a clinical sample, or that the questionnaire may not be as effective among such a young group of people.

The resilience constructs of pride and community connectedness do not appear to play a central role in the current studies referred adolescent group. This contrasts with various studies from the U.S. highlighting the importance of resilience factors, specifically pride and community connectedness (as suggested by Miller-Perusse et al. [28] & Wilson et al. [38]). It might be that Dutch clinic-referred TGD youth have different experiences compared to their U.S. counterparts, potentially due to cultural and social differences [44]. In cultures with higher acceptance and visibility of TGD individuals (such as the Netherlands) [71], the buffering effect of identity pride may be less pronounced compared to countries with TGD lower acceptance, where resilience is needed to protect against external stressors. However, although pride did not show direct protective associations with mental health outcomes, pride displayed negative edges with internalized transphobia and negative expectations for future events, both of which were positively associated with depression and internalizing problems, suggesting small indirect pathways through which resilience factors may relate to mental health outcomes. Investigating potential differences by country could inform intervention efforts to address the health needs of TGD youth living in different countries. Additionally, community connectedness showed a small positive association with non-affirmation. This finding, while seemingly counterintuitive, aligns with research such as the work of Begeny and Huo [72], who suggested that feeling valued within one’s minority group can enhance mental health but also increase perceptions of discrimination, which negatively impacts mental health. The relatively high level of non-affirmation within our clinical sample, compared to the other GMSR constructs, may result in these two factors mutually reinforcing one another. For example, TGD youth who are more engaged with supportive communities may become more attuned to instances where their identities are not affirmed in broader social contexts. This increased awareness could contribute to higher reported levels of non-affirmation.

Although not the primary focus of our study, 41.7% of Dutch referred TGD youth scored within the clinical range for YSR internalizing problems, which is notably higher than the 10% in the Dutch general population standardization sample [47] and proportions reported in studies conducted at the Center of Expertise on Gender Dysphoria (Amsterdam UMC) between 2008 and 2018 [8, 73]. The findings on the BDI-II (i.e., 32.7% had BDI-II scores within the range indicative of mild to severe depression) were similar to results from previous studies from our Center [40]. The elevated proportion on the YSR internalizing problems may be partly explained by the age composition of our sample, which included adolescents aged 13 and older; as shown by de Rooy et al. [73], older adolescents referred to gender identity services tend to report significantly more internalizing problems on the YSR than younger adolescents, suggesting that psychological difficulties may intensify with age.

In light of this vulnerability, the bridge scales of non-affirmation, internalized transphobia, and negative expectations for future events may be crucial for consideration within a clinical context. These nodes play bridging roles in linking other GMSR constructs to mental health outcomes. Addressing these bridge scales in clinical practice (i.e., deactivation in the network) could mitigate the influence of these other constructs in the network (e.g., distal stressors), thereby reducing their impact on mental health. Interventions could target these bridge scales and cultivate environments where individuals feel safe to express their gender identity. The three constructs can be addressed individually during clinical interventions but are also influenced by societal experiences. Therefore, societal-level interventions, including raising awareness about the value of affirming gender identity, are necessary [74].

Several key limitations of the present study should be noted. First, although network analysis can provide insights into potential connections, the cross-sectional nature of this study limits the ability to definitively establish causal relationships. Second, generalization of our findings to other groups of Dutch and non-Dutch TGD youth should be made with caution (e.g., non-clinical samples, youth from other countries/contexts, youth who have been excluded due to parental non-consent). Moreover, our sample was predominantly assigned female at birth, which limits the generalizability to trans youth assigned male at birth, whose minority stress processes may differ. Third, the operationalization of GMSR constructs may not have fully captured essential GMSR processes among TGD youth. While the GMSR measure posits consistent associations across variables within each domain (i.e., distal stress, proximal stress, resilience) [10], the inclusion of the discrimination and rejection constructs in the GMSR measure might reduce the overall score on distal stress. These constructs contain questions that may not be applicable to the assessed Dutch TGD youth (e.g., difficulties in finding housing and employment, rejection by an ethnic/racial community). The majority of the referred Dutch youth within our sample (99.2%) live with their parents. Thus, finding housing and/or employment may not be a current challenge among these referred youth. Additionally, next to assumed low face validity, Cronbach’s alpha of the gender-related discrimination and rejection constructs was quite low (0.56 and 0.52 respectively). These low internal consistency scores suggests that these constructs may not consistently measure the intended constructs, potentially impacting the replicability of the findings. However, Cronbach’s alpha can be depressed when scales include only a small number of items, so these values should not be interpreted as evidence of poor construct validity alone. Furthermore, although the GMSR measure has been translated into Dutch, its psychometric properties have not yet been formally validated, and therefore any cross-cultural differences should be interpreted with caution. Future research should aim to refine the constructs to improve their reliability and/or validity, possibly by including more relevant items or by revising the existing constructs to better capture the experiences of TGD youth. Fourth, the correlation stability coefficient (CS-C) is above the minimum threshold of 0.25 (CS-C = 0.285), indicating a moderate level of reliability, but it is not as robust as a coefficient above 0.5, which is deemed excellent. Increasing the sample size in future research would improve model stability and robustness. Finally, the study focused exclusively on constructs included in the GMSR measure and did not incorporate general psychological factors (e.g., coping) or additional resilience-related constructs (e.g., social and family support) that are also hypothesized to influence minority stress processes [75]. Future studies should consider incorporating a broader range of psychological factors and resilience-related constructs to provide a more comprehensive understanding of minority stress processes among TGD youth.

## Conclusion

This study provided the first evaluation of the associations between gender minority stress, resilience and mental health among clinic-referred Dutch TGD youth. Utilizing a network approach, the study provided insights into the complex interplay between various gender minority stressors and mental health, highlighting factors related to the psychological vulnerability of TGD youth. Key findings indicate that both distal (i.e., external) and proximal (i.e., internal) stressors are related to depression, internalizing problems, and suicidality, with non-affirmation exhibiting the highest strength centrality within the network. This suggests that non-affirmation plays a central role in the psychological network of gender minority stressors among our clinical sample of young adolescents, warranting further exploration in future research and offering targets for a clinical and societal approach. The study also revealed that resilience constructs such as pride and community connectedness do not play a central role in the mental health of our Dutch TGD youth sample, contrasting some findings from U.S.-based studies [28, 38, 44]. This discrepancy may be attributed to cultural differences between the Netherlands and U.S. in acceptance and visibility of TGD individuals, emphasizing the need for context-specific interventions. Non-affirmation, internalized transphobia, and negative expectations for future events were identified as critical constructs linking other gender minority stressors to mental health. Addressing these bridging constructs in clinical practice may mitigate the impact of the remaining stressors on mental health. Societal-level interventions, including raising awareness about the value of affirming gender identity and actively promoting gender affirmation within families, schools, and communities, are necessary to foster inclusive environments that reduce minority stress exposure and may be particularly impactful in lowering mental health problems among TGD youth.

## Supplementary Information

Supplementary Material 1

## Data Availability

The datasets generated for this study are available upon request from the corresponding author.

## Abbreviations

BDI-II: Beck Depression Inventory-II
CC: Community connectedness
CI: Confidence interval
CS-C: Correlation stability coefficient
D: Discrimination
DEP: Depression
EBIC: Extended Bayesian Information Criterion
GMSR: Gender Minority Stress and Resilience
INT: Internalizing problems
IT: Internalized transphobia
LCL: Lower confidence limit
NBGQ: Non-binary and genderqueer
ND: Non-disclosure of gender identity/history
NFE: Negative expectations for future events
P: Pride
R: Rejection
S: Suicidality
SD: Standard deviation
TGD: Transgender and gender-diverse
UCL: Upper confidence limit
V: Victimization
YSR: Youth Self-Report
