# Supplementary material for: Gender minority stress, resilience, and mental health in clinic-referred transgender and gender-diverse adolescents: a network analysis
Source: Child Adolesc Psychiatry Ment Health. 2026 Jan 29;20:27. doi: 10.1186/s13034-026-01031-6 (PMC12930926; doi:10.1186/s13034-026-01031-6)
Supplement: Supplementary file 1 — Supplementary Material 1 [file 13034_2026_1031_MOESM1_ESM.docx]

Figure S1. Nonparametric bootstrapped difference tests

A:
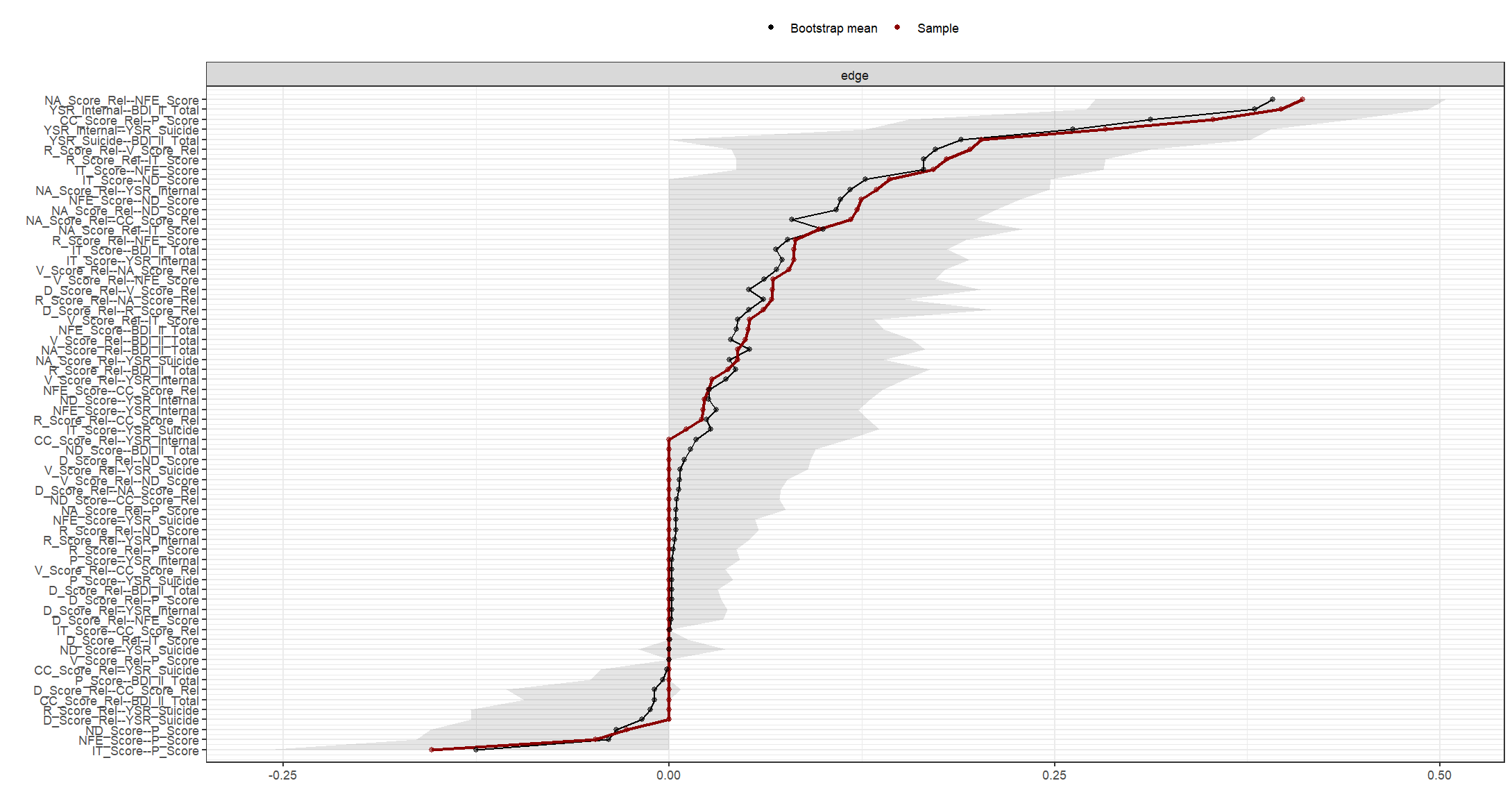


B:


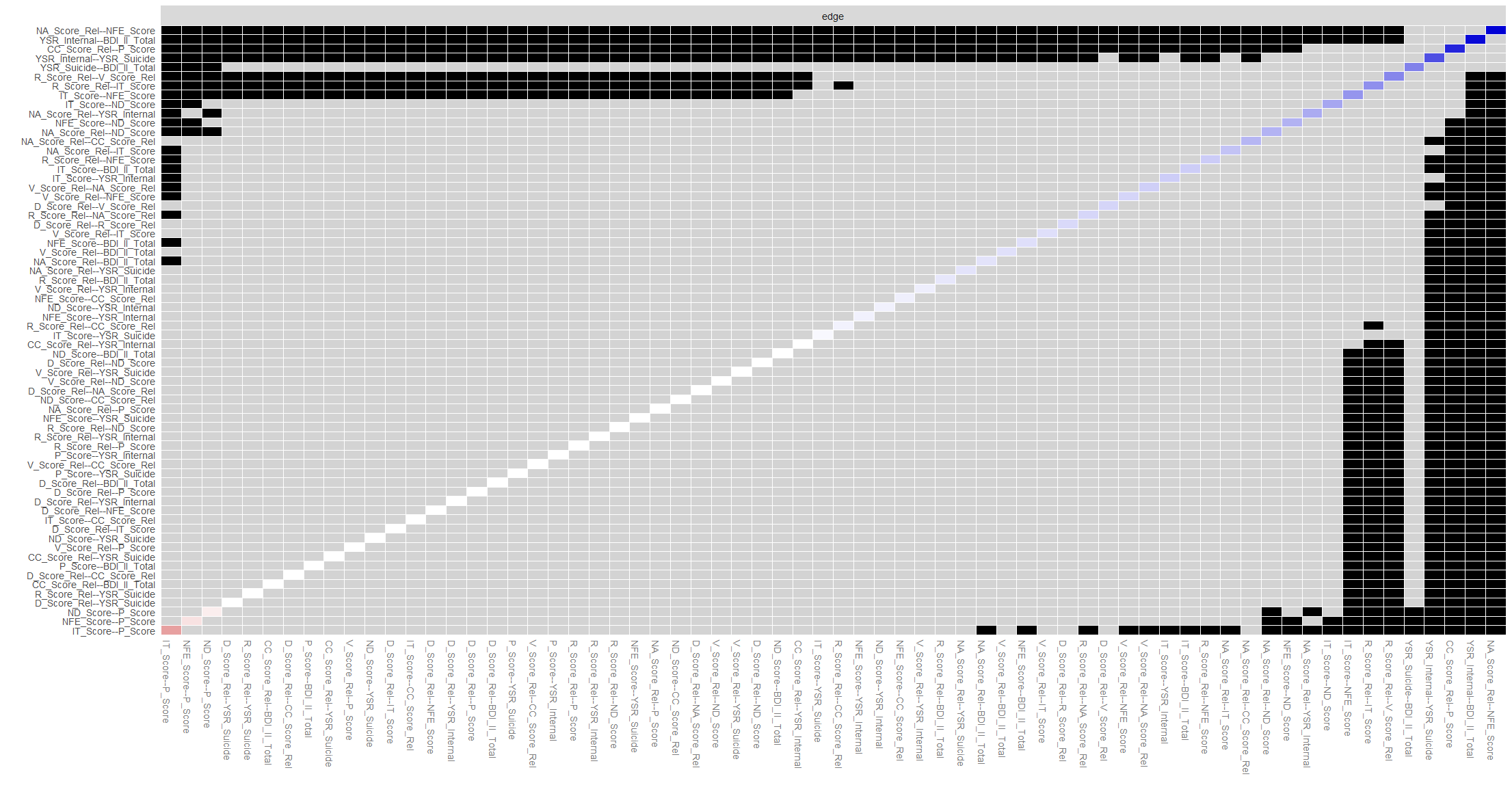


Figure S1. Panel A and Panel B: Nonparametric bootstrapped difference tests for the edges. For this, 95% bootstrapped confidence intervals (CIs) for edge-weights are constructed based on the normal variance in the bootstrapped sample. A wide interval represents low stability, and narrow intervals represent high stability.
